# Community Coalition Building and Human-Centered Design Strategies to Advance Homeless Health Systems: A Case Study from Rural North Carolina

**DOI:** 10.3390/ijerph23060784

**Published:** 2026-06-11

**Authors:** Ashley Jarrett, Oscar Fleming, Jacob Shomali, William Romani

**Affiliations:** 1 Burke County Public Health, Morganton, NC 28655, USA; 2 Gillings School of Global Public Health, University of North Carolina at Chapel Hill, Chapel Hill, NC 27599, USA; oscar.fleming@unc.edu (O.F.); jacob.shomali99@gmail.com (J.S.); 3 Innovate Carolina, University of North Carolina at Chapel Hill, Chapel Hill, NC 27514, USA; wromani@unc.edu

**Keywords:** rural health, systems thinking, street medicine, co-design, mobile health, community collaboration, health equity

## Abstract

**Highlights:**

**Public health relevance—How does this work relate to a public health issue?**

- Individuals experiencing homelessness in rural communities face persistent gaps in healthcare access due to fragmented systems and limited outreach infrastructure.
- This work examines how a rural county addresses these gaps through systems change rather than isolated service delivery models.

**Public health significance—Why is this work of significance to public health?**

- Demonstrates how coalition-based approaches can transform informal outreach into coordinated, sustainable health system responses.
- Provides practice-based evidence on integrating mobile health within rural safety-net systems.

**Public health implications—What are the key implications or messages for practitioners, policy makers and/or researchers in public health?**

- Supports the use of systems thinking, co-design, and cross-sector governance to improve healthcare access for marginalized populations.
- Identifies policy and financing strategies needed to sustain mobile and community-based care in rural settings.

**Abstract:**

In Burke County, North Carolina, a Hepatitis A outbreak among unsheltered residents exposed gaps in access to clinic-based care and prompted early, ad hoc “backpack medicine” outreach efforts to deliver care directly in nontraditional settings. While this approach addressed immediate needs, it highlighted the inadequacy of isolated interventions, prompting local partners to pursue more structured, coordinated, and community-driven approaches to homeless health system design. This project report describes how Burke County Public Health, in partnership with the University of North Carolina at Chapel Hill, applied systems thinking, community coalition building, and human-centered design to transition from reactive outreach to a structured, sustainable mobile health delivery model for people experiencing homelessness. Guided by Community Coalition Action Theory (CCAT), partners used human-centered design methods to engage over 40 community stakeholders and 10 individuals with lived experience of homelessness or housing instability. Through empathy mapping, iterative prototyping, and thematic analysis, the team identified priority service gaps, defined operational requirements, and developed prototype service models, while building cross-agency readiness for implementation.

## 1. Introduction

Rural counties in the United States face persistent challenges in delivering equitable healthcare due to limited financial resources, workforce shortages, and infrastructural constraints [1]. These challenges are further compounded by geographic isolation, transportation barriers, and fragmented safety net systems, which can limit access to care for people experiencing homelessness (PEH) [2]. Mobile health interventions, including street medicine, can address these gaps, reaching individuals who are unable or unlikely to engage in clinic-based care. Yet many of these programs remain vulnerable when they depend primarily on volunteer labor, short-term funding, or informal partnerships. Recent evidence suggests that healthcare access for PEH is strengthened by continuity of care, integrated service delivery, and cross-sector collaboration, highlighting the importance of coordinated governance structures that extend beyond individual outreach efforts [2,3].

Burke County in western North Carolina is a largely rural area spanning approximately 506 square miles with a population of 88,655 [4]. Like many rural Appalachian communities, the county faces economic and infrastructural conditions that shape access to care, including lower median household income, limited transportation infrastructure, and a fragmented healthcare safety net. Nearly 14% of residents live below the federal poverty line. Educational and employment opportunities lag state averages, contributing to increased vulnerability among medically underserved populations [4].

Housing instability and homelessness have become increasingly visible concerns within Burke County. The 2025 North Carolina Balance of State Continuum of Care Point-in-Time Count identified 304 individuals experiencing homelessness in the county, including 46 individuals living in unsheltered settings [5]. Rural patterns of homelessness, characterized by dispersion across encampments, wooded areas, vehicles, and other informal sheltering arrangements, can complicate outreach, continuity of care, and service coordination [6]. Mental and physical health issues, and related costs, are key drivers of homelessness which typically further undermines health outcomes [6,7]. Available health services in the county remain largely clinic-based and geographically centralized, creating barriers for individuals living outdoors or in unstable housing. In this context, effective public health responses require approaches that operate across organizational and jurisdictional boundaries, making coalition-based and community-engaged strategies essential for feasibility, reach, and sustainability [8].

The county’s public health system historically operated within a constrained safety net ecosystem comprising the local health department, a free and charitable clinic, a federally community health center, faith-based organizations, and emergency services. These entities often functioned in parallel rather than in coordination, resulting in duplicated efforts, inconsistent follow-up, and limited reach to individuals living outside traditional service settings. Similar patterns have been documented in other rural health systems, where fragmented service delivery can limit access and continuity of care [9].

When Burke County experienced a Hepatitis A outbreak in 2021, these structural limitations became apparent in real time. Public health officials faced challenges in locating, vaccinating, and following individuals experiencing homelessness due to the absence of shared data systems and outreach infrastructure. In response, local partners mobilized to deliver vaccines and basic care directly to unsheltered residents through informal coordination among public health staff, volunteer clinicians, outreach workers, and community organizations. As outbreak response activities evolved, these efforts developed into a semi-structured “backpack medicine” approach in which primary care, wound care, and preventive services were delivered directly to encampments and other nontraditional settings. This model functioned for approximately 18 months as the primary mechanism for reaching individuals disconnected from clinic-based care.

While backpack medicine improved access and facilitated trust-building with unsheltered residents, it relied heavily on individual relationships, volunteer capacity, and informal workflows rather than formalized governance structures and standardized processes. These limitations demonstrated that outreach alone was insufficient to support sustained engagement in and continuity of care. At the same time, these efforts revealed the potential of cross-sector collaboration and community-informed approaches to reach underserved populations. As outbreak response transitioned into recovery, these lessons informed the development of the Burke County Homeless Health Initiative, a structured, community co-designed model focused on systems change, shared responsibility, and long-term sustainability.

This manuscript presents a retrospective qualitative project report describing the evolution of the Burke County Homeless Health Initiative from outbreak response to a structured, community co-designed mobile health model. The report focuses on systems development, coalition formation, and implementation learning within a rural safety net setting and contributes practice-based evidence to inform similar efforts in resource-limited communities.

## 2. Materials and Methods

### 2.1. Project Design and Approach

This project employed a qualitative retrospective case study design to examine the development of a rural, cross-sector initiative to improve healthcare access for people experiencing homelessness in Burke County, North Carolina. The initiative was designed to move beyond isolated outreach activities toward a coordinated system capable of supporting sustained engagement, continuity of care, and shared responsibility across participating organizations. The analysis focused on organizational learning, coalition development, community engagement, and service co-design activities conducted between 2021 and 2025. The purpose of the project was to understand how systems-level collaboration and human-centered design contributed to the development of a coordinated homeless health initiative within a rural safety-net setting.

Three complementary frameworks guided the work. Systems Thinking informed efforts to understand the relationships among organizations, services, and individuals within the local safety-net ecosystem and to identify factors influencing healthcare access and coordination. Community Coalition Action Theory (CCAT) provided a framework for coalition formation, collaborative leadership, and shared governance among participating organizations. Human-Centered Design (HCD) served as the primary approach for stakeholder engagement, needs assessment, prototype development, and iterative refinement of potential service models. Human-centered design activities included community immersion, focus groups, stakeholder workshops, empathy mapping, and iterative prototype development. Participants included healthcare providers, social service agencies, faith-based organizations, law enforcement, and individuals with lived experience of homelessness. Two rounds of co-design sessions were conducted. The first explored barriers and opportunities related to healthcare access and generated potential service concepts. The second obtained participant feedback on prototype service models and informed refinement and selection of a pilot intervention.

The project was led by Burke County Public Health in partnership with the University of North Carolina at Chapel Hill. Beginning in 2024, activities were supported by the Translating Innovative Ideas for the Public Good (TIIP) Award through Innovate Carolina, which provided resources to conduct a structured co-design process focused on improving healthcare access for individuals experiencing homelessness.

### 2.2. Stakeholder Engagement and Co-Design Activities

Data for the cumulative project report derived from multiple sources generated throughout the development of the Burke County Homeless Health Initiative between 2021 and 2025. These sources included coalition meeting records, planning materials, interviews and focus groups, co-design artifacts, internal communications among participating organizations, implementation reports, and other project documentation. Together, these materials provided a comprehensive account of how partnerships, governance structures, stakeholder priorities, and service delivery strategies evolved over time within the local safety-net system.

Human-centered design activities were conducted between July 2024 and early 2025 and engaged a diverse group of stakeholders representing healthcare organizations, social service agencies, faith-based organizations, emergency services, public health, and individuals with lived experience of homelessness or housing instability. Prior to formal data collection, project team members participated in a period of community immersion intended to strengthen relationships and better understand local experiences of homelessness. Team members attended community meal services, engaged in informal discussions with residents and service providers, and visited local encampments with the assistance of trusted community partners. These activities provided contextual understanding of healthcare barriers, service experiences, and community priorities and informed the development of subsequent focus group guides and co-design activities.

Over 40 formal stakeholder engagements occurred through a series of focus groups, interviews, coalition meetings, and facilitated design workshops. Initial sessions engaged healthcare providers, social service agencies, outreach organizations, law enforcement representatives, and other community stakeholders in discussions regarding barriers to healthcare access and opportunities for service improvement. Participants completed design exercises and systems-mapping activities intended to identify gaps within the existing safety-net system and generate potential solutions.

10 individuals with lived experience of homelessness participated through focus groups and informal interviews. Women were engaged through an established support group hosted by a local community organization. Although a dedicated focus group for men was planned, participation was limited, resulting in the use of individual interviews conducted within community settings. Discussions explored healthcare experiences, barriers to care, preferences for service delivery, and factors influencing engagement with mobile health services.

Human-centered design tools including empathy mapping, journey mapping, systems mapping, and rapid prototyping were used throughout the process. Feedback from stakeholders informed the development of two service prototypes characterized as an “on-foot” outreach model and an “on-wheels” mobile clinic model. During subsequent co-design sessions, participants reviewed, refined, and evaluated both concepts. Feedback from individuals with lived experience directly informed prototype selection and implementation planning.

### 2.3. Analytical Framework and Synthesis

Systems Thinking served as the primary lens for understanding how organizational relationships, service structures, and community conditions interacted over time to influence healthcare access for people experiencing homelessness. Homelessness is increasingly recognized as a complex adaptive challenge shaped by interactions among healthcare systems, social services, community organizations, policies, and social determinants of health rather than by any single determinant [10]. Systems approaches emphasize understanding relationships, information flows, interdependencies, feedback processes, and organizational adaptation across sectors that collectively influence outcomes [11]. Accordingly, analysis focused on identifying system actors, structural barriers, information-sharing processes, feedback mechanisms, and adaptive responses that emerged throughout initiative development. Particular attention was given to how changes in one component of the local safety-net system influenced other organizations and contributed to broader system adaptation.

Data were analyzed using a qualitative thematic synthesis approach informed by Systems Thinking and Community Coalition Action Theory (CCAT). Thematic analysis was selected because it provides a flexible and rigorous method for identifying, organizing, and interpreting patterns across diverse qualitative data sources while allowing examination of complex organizational and community processes. Consistent with Braun and Clarke’s approach to thematic analysis, analysis involved iterative engagement with project materials to identify recurring patterns, relationships, and meanings relevant to the development of the Burke County Homeless Health Initiative [12].

Community Coalition Action Theory complemented this perspective by providing a framework for examining coalition formation, collaborative capacity, leadership development, governance structures, partnership development, and coalition outcomes. CCAT recognizes that coalition functioning is influenced by community context, existing organizational relationships, and collaborative processes that evolve over time [13]. The framework informed interpretation of how participating organizations moved from informal outbreak-response collaboration toward a more coordinated system characterized by shared governance, collective problem solving, and cross-sector engagement.

Project materials were reviewed iteratively using procedures consistent with thematic analysis. Data sources included project documentation, coalition meeting records, planning materials, focus groups and interviews, co-design artifacts, implementation reports, internal communications, and evaluation materials generated between 2021 and 2025. Initial review focused on familiarization with project records and identification of meaningful events, decisions, recurring challenges, and adaptation processes. Subsequently, project materials were coded and organized into thematic categories related to healthcare access, coalition development, stakeholder engagement, governance, service innovation, implementation learning, and sustainability. Themes were continuously reviewed, refined, and compared across data sources to identify convergence and divergence across stakeholder perspectives and implementation experiences.

As analysis progressed, findings were organized chronologically and synthesized into four implementation phases representing distinct periods of learning, adaptation, coalition development, and systems change. Phase identification was informed by major shifts in organizational relationships, governance structures, stakeholder engagement processes, service delivery strategies, and coalition maturity. The resulting synthesis was used to develop an integrated understanding of both system-level and coalition-level change throughout the evolution of the Burke County Homeless Health Initiative.

### 2.4. Logic Model and Timeline Development

To enhance clarity and transparency in the presentation of a multi-year systems change initiative, a logic model and chronological timeline were developed during the later stages of analysis and synthesis. These tools functioned as analytic outputs rather than guiding frameworks and were derived from themes, implementation activities, and developmental milestones identified through the thematic synthesis process. Logic models are commonly used to visually depict relationships among program inputs, activities, and outcomes and to communicate how complex initiatives are expected to achieve change over time.

The logic model was used to represent relationships among coalition resources, stakeholder engagement activities, implementation strategies, and anticipated system-level outcomes, while the timeline illustrated the sequence of major events, adaptation processes, and implementation phases that contributed to the evolution of the Burke County Homeless Health Initiative. Consistent with the systems-oriented approach used throughout this project, these visual tools helped depict how organizational relationships, coalition development, community engagement, and service innovations evolved in response to emerging needs, opportunities, and external events [14].

The development of these tools supported interpretation of complex interactions across project phases by providing a structured representation of how activities, partnerships, and outcomes evolved over time. Similar approaches have been used in public health evaluation to synthesize diverse sources of information, facilitate stakeholder understanding, and communicate the theory of change underlying complex initiatives. As such, the logic model and timeline served as complementary tools for organizing and communicating findings from a practice-based initiative that developed through iterative learning, adaptation, and cross-sector collaboration over multiple years.

### 2.5. Ethical and Regulatory Considerations

This project was conducted as a public health practice initiative focused on program implementation, organizational learning, coalition development, and service planning rather than as human subjects research. The primary purpose of the work was to strengthen healthcare access for people experiencing homelessness through community engagement, systems improvement, and collaborative service development. Accordingly, the analysis focused on organizational processes, implementation activities, coalition development, and systems-level change rather than on individual participants or clinical outcomes.

Throughout the initiative, individuals with lived experience of homelessness participated in community engagement activities, including interviews, focus groups, and co-design sessions intended to inform service planning and improvement. These activities were conducted as part of routine program development and quality improvement efforts and were not designed to test research hypotheses or generate generalizable knowledge. No identifiable personal information, individual-level clinical data, or protected health information were collected for the purposes of this analysis.

The University of North Carolina determined that institutional review board review was not required because the project constituted public health practice and organizational learning rather than human subjects research. All materials included in this report were derived from routine public health operations, coalition activities, quality improvement efforts, implementation records, and collaborative planning processes. Findings are presented in aggregate and focus on systems-level observations and implementation experiences rather than individual participant perspectives.

## 3. Implementation Phases

**Phase 1: Outbreak Response and Recognition of System Gaps (2021)**

In 2021, Burke County’s public health system responded to a Hepatitis A outbreak affecting individuals experiencing homelessness. Outreach efforts focused on vaccine delivery, case identification, and follow-up among unsheltered populations and required coordination across the local health department, emergency medical services, clinics, shelters, and community organizations. As response activities expanded, teams encountered persistent operational barriers that limited the effectiveness of traditional public health approaches, including difficulty locating individuals living outdoors, limited transportation capacity, the absence of shared data systems, and inconsistent follow-up mechanisms. These constraints resulted in reactive, labor-intensive outreach processes and limited the ability to ensure continuity of care.

These conditions revealed critical structural gaps in the local safety-net system, including the absence of coordinated mobile care infrastructure and formal mechanisms to support cross-sector collaboration. Although the outbreak response temporarily strengthened communication across organizations, these connections were informal and ad hoc. As response efforts stabilized, partners recognized that existing systems were not designed to reach unsheltered populations and that system-level redesign would be required to address persistent access barriers.

**Phase 2: Pilot Backpack and Street Medicine Intervention (2021–2023)**

Following the Hepatitis A outbreak, a coalition of volunteer clinicians, public health staff, and outreach workers continued providing care through a semi-structured backpack medicine approach. Teams delivered primary care, wound care, vaccinations, and screenings directly in encampments and other nontraditional settings. Over time, outreach routes became more consistent and trust developed between providers and unsheltered residents. Informal partnerships strengthened among emergency medical services, the free clinic, shelters, and faith-based organizations, improving short-term referral pathways and facilitating initial linkage to care. This phase marked the first sustained effort to deliver healthcare outside traditional clinical environments in Burke County.

Despite these advances, the absence of formal infrastructure constrained scalability and consistency. Clinical supplies varied across outreach events, documentation practices were inconsistent, and liability coverage and data-sharing processes remained unresolved. The model relied heavily on volunteer labor and individual relationships rather than standardized workflows or shared governance structures. As resource constraints intensified and participation fluctuated, the model’s dependence on individual actors instead of system-level integration became increasingly apparent. By late 2023, partners recognized that while backpack medicine improved access and engagement, it could not support sustained system-level impact without formal infrastructure, coordinated governance, and institutional alignment.

**Phase 3: Systems Thinking, Infrastructure Expansion, and Disaster Preparedness (2023–2024)**

In response to the limitations identified during earlier phases, partners began transitioning from informal outreach toward a systems-oriented approach to improving healthcare access for individuals experiencing homelessness. Rather than focusing solely on expanding direct services, stakeholders sought to better understand how existing organizations, resources, and programs interacted within the local safety-net system. Through ongoing collaboration, it became increasingly apparent that many organizations were serving the same population while operating independently, resulting in fragmented communication, inconsistent referral pathways, and limited coordination across sectors.

Burke County Public Health assumed a central convening role and worked to strengthen relationships among healthcare providers, social service organizations, faith-based groups, emergency services, law enforcement, and community partners. The goal was not to create a new standalone program but to improve alignment among existing resources and establish a shared understanding of community needs. During this period, an Academic Health Department partnership was established with the Gillings School of Global Public Health at the University of North Carolina at Chapel Hill to support implementation learning, workforce engagement, and structured reflection on system development. This partnership provided additional capacity to document lessons learned and translate field-based experience into future planning efforts.

As relationships strengthened, outreach activities became increasingly integrated into broader community systems. Mobile outreach efforts were incorporated into Code Purple winter shelter operations, encampment clean-up initiatives, and emergency preparedness planning. These partnerships proved particularly valuable during periods of severe weather and disaster response, creating opportunities for organizations that had historically worked independently to coordinate around shared challenges. By the end of this phase, the initiative had evolved from a volunteer-driven outreach effort into a more organized network characterized by regular communication, shared problem solving, and growing accountability among participating organizations. This foundation created the conditions necessary for the structured co-design process that followed.

**Phase 4: Human-Centered Design, TIIP Award, and Women’s Health Pilot (2024–2025)**

By 2024, partners recognized that improving healthcare access would require more than organizational coordination alone. Although service providers had identified numerous barriers to care, there was growing recognition that individuals experiencing homelessness needed a more direct role in shaping future solutions. The Translating Innovative Ideas for the Public Good (TIIP) award from Innovate Carolina provided resources to support a structured human-centered design process focused on developing community-informed strategies for improving healthcare access.

Implementation of the co-design process emphasized relationship building and trust development before formal planning activities began. Project leaders spent time in community settings where individuals experiencing homelessness already gathered, including meal programs and other service locations, engaging residents in informal conversations about healthcare experiences, service barriers, and unmet needs. These interactions helped establish trust, improve understanding of local context, and create opportunities for participation among individuals who may have been reluctant to engage in more formal planning processes.

As engagement activities progressed, individuals with lived experience participated alongside healthcare providers, social service organizations, outreach teams, and community partners in shaping future service concepts. Rather than simply providing feedback on predetermined ideas, participants helped identify priorities, challenge organizational assumptions, and influence the direction of service development. Women experiencing homelessness or housing instability consistently described unmet needs related to reproductive healthcare, preventive screenings, prenatal care, and access to services delivered in environments perceived as safe, respectful, and nonjudgmental. These perspectives broadened organizational understanding of healthcare access barriers and shifted conversations toward opportunities that were not initially prioritized by participating agencies.

The iterative nature of the co-design process enabled community feedback to directly influence subsequent planning activities. Early discussions informed the development of multiple mobile service concepts, while later engagement activities allowed participants to evaluate, refine, and compare potential approaches. Through this process, stakeholders identified a women’s health-focused mobile service model as both a high-priority community need and a feasible pilot opportunity. The resulting Women’s Health Mobile Unit Pilot Program reflected not only organizational priorities but also the expressed preferences and experiences of individuals who would ultimately use the service.

The pilot was launched in late 2025 through a partnership among Burke County Public Health, Good Samaritan Clinic, UNC Health Blue Ridge, Burke United Christian Ministries, and the UNC Gillings School of Global Public Health. A mobile medical unit was deployed at the Burke United Christian Ministries service center during an eight-week pilot period. Services included reproductive healthcare, preventive screenings, sexually transmitted infection testing, contraceptive counseling, and wellness visits delivered using trauma-informed and culturally responsive approaches. The primary purpose of the pilot was to assess feasibility, partnership workflows, and service acceptability rather than clinical outcomes.

Beyond testing a mobile service model, the pilot generated important implementation insights regarding partnership coordination, community engagement, and mobile healthcare delivery in a rural setting. The process demonstrated how structured co-design can support service innovation while strengthening relationships among organizations and community members. It also reinforced the value of sharing decision-making with individuals who possess lived experience of the challenges the initiative sought to address.

## 4. Results

The phased development of the Burke County Homeless Health Initiative produced documented process and implementation outcomes related to coalition development, community-informed service design, pilot implementation, and systems-level learning. These findings do not represent clinical, longitudinal, or population-level health outcomes. Rather, they describe changes observed through project documentation, coalition records, co-design materials, implementation reports, and partner reflection. Collectively, the findings demonstrate how the initiative moved from fragmented outreach activities toward a more coordinated and adaptive approach to homeless health within the local safety-net environment.

### 4.1. Coalition Development and Cross-Sector Collaboration

A primary process outcome of the initiative was the development of a sustained cross-sector coalition focused on improving healthcare access for people experiencing homelessness. Analysis of coalition records, planning materials, stakeholder engagement activities, and implementation documents demonstrated increasing participation across healthcare, public health, social services, emergency services, community organizations, academic partners, and individuals with lived experience of homelessness. More than 40 community stakeholders and approximately 10 individuals with lived experience participated in interviews, focus groups, co-design workshops, planning sessions, and implementation activities throughout the project period.

Coalition development was reflected not only in expanding membership but also in increasing collaboration across organizations that had historically operated independently. Participating organizations jointly contributed to outreach planning, co-design activities, emergency preparedness efforts, pilot implementation, and evaluation activities. Evidence of collaboration included coordinated participation in Code Purple shelter planning, encampment outreach efforts, mobile health pilot development, and implementation of the Women’s Health Mobile Unit pilot. These activities created opportunities for shared problem-solving and facilitated communication among organizations that previously interacted only episodically.

Over time, participating agencies began moving from parallel service delivery toward more coordinated approaches to outreach, referral planning, and service development. Although formal governance structures remained under development, coalition activities generated growing consensus regarding the need for shared accountability, coordinated decision-making, and sustainable infrastructure to support future homeless health initiatives. These findings represent coalition development and organizational process outcomes rather than evidence of health impact.

### 4.2. Development of Community-Informed Service Strategies

A second major finding was the identification of community priorities through structured co-design activities. Engagement sessions involving service providers, community organizations, public safety personnel, and individuals with lived experience consistently highlighted the importance of trust, relationship-building, accessibility, and continuity of engagement in healthcare delivery. Stakeholders frequently emphasized that healthcare access barriers extended beyond clinical services and included transportation limitations, stigma, fragmented service systems, and difficulty navigating available resources.

Importantly, individuals with lived experience influenced not only problem identification but also solution development. Feedback from community participants informed the refinement of service concepts throughout the co-design process and contributed directly to subsequent planning decisions. The process generated two mobile service prototypes: an “on-foot” outreach model emphasizing flexibility and direct engagement in community settings and an “on-wheels” model centered on a mobile clinical unit capable of delivering a broader range of services. Through iterative review and feedback, participants identified strengths and limitations of each approach and helped inform prototype refinement.

Gender-specific engagement activities revealed unique concerns related to women’s health services that had not been fully recognized by participating organizations. Women described challenges accessing reproductive healthcare, preventive screenings, and other gender-specific services while experiencing homelessness or housing instability. These findings shifted stakeholder priorities and contributed to selection of a women’s health-focused mobile service model for pilot implementation. The resulting service concept reflected both organizational expertise and community-identified needs, demonstrating the practical value of shared decision-making within the co-design process.

### 4.3. Pilot Testing of a Mobile Women’s Health Service Model

The Women’s Health Mobile Unit pilot generated implementation findings related to feasibility, acceptability, appropriateness, and adoption. The pilot was intentionally designed as an operational test of partnership workflows, service delivery processes, and cross-sector coordination rather than an evaluation of clinical effectiveness. Findings from the implementation evaluation are summarized in Table 1 and informed interpretation of pilot performance, operational challenges, and opportunities for future service development.

The pilot was conducted between October and December 2025 through a partnership among Burke County Public Health, UNC Health Blue Ridge, Good Samaritan Clinic, Burke United Christian Ministries, and the University of North Carolina Gillings School of Global Public Health. Planned implementation included 16 service days and engagement of 35 women. Actual implementation resulted in 10 service days and services delivered to eight women, while all five core partner organizations remained actively engaged throughout planning, implementation, troubleshooting, and evaluation activities. Three men who approached the service seeking assistance were referred to other available healthcare resources.

Implementation findings demonstrated that the model was generally acceptable and appropriate among participating clients and providers. Women who utilized the service reported appreciation for the availability of care, the services received, and the supplemental resources provided through the program. Providers consistently described the mobile women’s health model as acceptable and feasible within the local context. Evaluation findings further suggested that trust, consistency, and delivery of services in familiar, non-clinical environments were important contributors to engagement. The pilot also identified several implementation challenges relevant to future service development. Service reach was limited by provider availability, weather-related cancellations, delayed operationalization of the host site, transportation barriers, and the timing of service delivery relative to client availability. Community awareness of the service was reported as moderate to low, suggesting that future efforts may require more intensive outreach and marketing strategies. Additionally, delays in funding processes limited access to planned laboratory services during much of the pilot period.

Despite these challenges, participating organizations successfully coordinated staffing, registration, transportation, interpretation services, clinical care, and referral processes across agencies. Providers reported that standard women’s health services could be delivered within the mobile setting and that operational challenges were largely related to workflow and infrastructure rather than clinical feasibility. Evaluation findings also identified opportunities to strengthen communication among partners, improve data-sharing processes, develop security protocols, and align service schedules more closely with community needs. Participant and partner feedback suggested that the service model was acceptable and appropriate within the local context. Women were more willing to engage when services were delivered in familiar, non-clinical environments perceived as safe, respectful, and nonjudgmental. Trust and consistency were repeatedly identified as factors influencing engagement and were often viewed as equally important as the clinical services themselves.

The pilot suggested that mobile healthcare delivery was feasible while revealing implementation challenges common to rural mobile healthcare delivery. Participating organizations successfully coordinated staffing, clinical workflows, transportation, registration processes, and service delivery across agencies. At the same time, providers identified challenges related to scheduling, outreach, technology limitations, administrative processes, and delayed access to laboratory services. Despite modest service utilization, partners viewed the pilot as a valuable opportunity to test operational assumptions and identify infrastructure needs necessary for future service expansion. Future expansion will require stronger infrastructure, enhanced outreach strategies, and greater operational integration across participating organizations.

### 4.4. Systems-Level Learning

Beyond pilot implementation, the initiative generated systems-learning outcomes that informed ongoing planning and future development efforts. Analysis of implementation experiences consistently identified the need for stronger infrastructure to support sustainable mobile healthcare delivery. Key priorities included development of formal referral pathways, standardized documentation workflows, shared governance structures, sustainable funding mechanisms, and improved coordination across participating organizations.

The initiative also contributed to changes in how participating organizations conceptualized responsibility for healthcare access among people experiencing homelessness. Rather than viewing outreach and service delivery as the responsibility of a single organization, partners increasingly recognized homelessness as a shared community challenge requiring coordinated action across healthcare, public health, social services, emergency response, and community organizations. This shift was reflected in growing interest in formal coalition governance, long-term sustainability planning, and integration of mobile health efforts into broader community systems.

One indicator of this evolving commitment was the expansion of partner engagement following completion of the pilot. Organizations that had participated in coalition activities began exploring new opportunities to integrate homeless health priorities into their own operational and strategic planning efforts. For example, a local federally qualified health center subsequently partnered with Burke County Public Health to pursue external funding through the Street Medicine Institute to support development of a formal street medicine program. This effort reflected growing recognition that healthcare access for people experiencing homelessness should be incorporated into existing healthcare delivery systems rather than addressed solely through short-term pilot projects or volunteer outreach efforts.

Participating organizations also identified the need to move beyond informal collaboration toward more durable structures capable of supporting shared learning, decision-making, and sustainability. Discussions emerging from the pilot and coalition activities increasingly focused on formal governance models, leadership development, and mechanisms for coordinating activities across organizations while preserving local flexibility. Several partners expressed interest in exploring approaches consistent with Communities of Practice and other collaborative frameworks that could facilitate ongoing learning, workforce development, resource sharing, and coordinated action around homeless health and mobile healthcare delivery.

At the organizational level, the initiative highlighted the importance of institutionalizing homeless health work through policies, procedures, and operational priorities rather than relying solely on individual champions. Participating agencies recognized that sustained progress would likely require incorporation of homeless health considerations into strategic planning, workforce development, referral protocols, emergency preparedness activities, and community partnership structures. These findings reinforced that long-term sustainability depends not only on service delivery models but also on organizational commitment and policy alignment that support continued attention to the needs of individuals experiencing homelessness.

Finally, the project demonstrated the role of flexible planning and catalytic funding in supporting rural systems change. Stakeholders identified coalition development, co-design activities, operational planning, and pilot testing as critical precursors to sustainable service expansion. Rather than producing definitive evidence regarding health outcomes, the initiative generated practical knowledge regarding partnership development, governance, service design, and infrastructure requirements necessary to support future homeless health initiatives in rural communities. The work also created a platform for subsequent funding opportunities, service development efforts, and cross-sector partnerships that extend beyond the scope of the original pilot.

## 5. Discussion

The Burke County Homeless Health Initiative demonstrates how rural communities can move from fragmented outreach activities toward coordinated systems-level approaches for improving healthcare access among people experiencing homelessness. Rather than emerging from a single intervention or organizational strategy, the initiative evolved through multiple phases of learning, adaptation, coalition development, community engagement, and pilot implementation. This progression reflects the reality that complex health and social challenges in rural communities often require iterative systems development rather than isolated programs or short-term service expansions.

A central finding of this initiative was that sustainable progress depended less on the implementation of a specific mobile health service and more on strengthening relationships, governance structures, and operational infrastructure across the local safety-net system. Early outreach efforts demonstrated that healthcare could be successfully delivered outside traditional clinical settings and that trust could be established with individuals experiencing homelessness. However, these activities also revealed significant system limitations, including fragmented referral pathways, inconsistent communication processes, limited coordination across organizations, and heavy reliance on volunteer labor and individual champions. Similar challenges have been documented throughout the street medicine and mobile health literature, where promising outreach efforts frequently encounter barriers related to sustainability, coordination, and integration with broader healthcare systems. These findings suggest that mobile healthcare models alone may be insufficient unless accompanied by broader efforts to strengthen the systems within which they operate.

The initiative also illustrates the value of systems thinking as a practical approach to community health improvement. Systems mapping and coalition development activities helped stakeholders move beyond individual programs and organizations to better understand the interdependencies shaping healthcare access among people experiencing homelessness. Earlier phases of the initiative revealed that many organizations were serving the same population while operating largely independently, resulting in duplicated efforts, service gaps, and limited continuity of care. Through structured collaboration, stakeholders developed a more comprehensive understanding of how healthcare providers, public health agencies, social service organizations, emergency responders, faith-based groups, and community members collectively influenced access to care. This broader perspective shifted discussions away from isolated organizational solutions and toward shared responsibility for addressing homelessness as a community health challenge.

Human-centered design further contributed to this transition by creating structured opportunities for individuals with lived experience to participate in identifying service gaps, shaping priorities, and evaluating potential solutions. Rather than functioning solely as participants or informants, individuals experiencing homelessness influenced the direction of the initiative by helping identify unmet needs, evaluating prototype concepts, and informing the selection of the Women’s Health Mobile Unit pilot. These findings support growing evidence that co-design approaches can improve the relevance, acceptability, and contextual fit of community health interventions by incorporating experiential knowledge into decision-making processes. Within Burke County, the co-design process also served as a mechanism for organizational learning by helping participating agencies better understand the lived realities, priorities, and barriers experienced by individuals navigating homelessness in a rural environment.

The Women’s Health Mobile Unit pilot generated important implementation findings regarding feasibility, acceptability, appropriateness, and systems integration. Women who engaged with the service consistently emphasized the importance of trust, consistency, and receiving care in familiar community settings. Providers and partner organizations viewed the model as acceptable and operationally feasible, while also identifying barriers related to staffing, outreach, transportation, scheduling, laboratory access, and administrative infrastructure. Importantly, the pilot functioned less as a test of clinical effectiveness and more as a test of the system’s ability to coordinate services across organizational boundaries. The resulting implementation findings helped identify infrastructure requirements necessary for future service expansion, including referral pathways, governance mechanisms, data-sharing processes, funding strategies, and operational workflows.

Several findings from the Burke County initiative align with emerging systems-oriented approaches to homelessness and health reported elsewhere. For example, the Chicago Department of Public Health’s Systems Change Collaborative brought together healthcare providers, homeless service organizations, public agencies, advocates, and individuals with lived experience to identify system-level opportunities for improving health outcomes among people experiencing homelessness [15]. Similarly, London’s Whole of Community System Response to Health and Homelessness engaged more than 70 organizations and over 200 leaders in a coordinated effort to redesign community responses through shared governance, collective action, and cross-sector accountability [16]. Likewise, the Secure Jobs Initiative demonstrated how systems thinking and network development can help communities address complex social challenges by building collaborative infrastructure, strengthening interorganizational relationships, and leveraging existing community resources rather than relying solely on new programs or services [17]. Collectively, these examples support the growing recognition that systems-level approaches may be essential for addressing the complex and interconnected factors contributing to homelessness and poor health outcomes.

At the same time, the Burke County initiative differs from many previously described efforts in several important ways. First, the initiative emerged from a communicable disease outbreak response rather than from a healthcare organization, housing agency, or formal planning process. Second, the work evolved within a rural Appalachian context characterized by geographic dispersion, transportation barriers, limited healthcare infrastructure, and a relatively small but highly vulnerable population of individuals experiencing homelessness. Third, the initiative progressed beyond coalition development and systems analysis to include community co-design, prototype development, pilot implementation, and subsequent infrastructure development activities. This progression provides a practical example of how rural communities can move from recognizing a problem to building the relationships, governance structures, and implementation capacity necessary to support long-term systems change.

The findings also highlight an important shift in how participating organizations conceptualized responsibility for homelessness and health. Over time, coalition members increasingly recognized that improving healthcare access for people experiencing homelessness could not be delegated to any single organization, outreach team, or service sector. Instead, responsibility became distributed across healthcare providers, public health agencies, social service organizations, emergency response systems, academic institutions, faith-based organizations, and community partners. This shift mirrors observations from other systems-change initiatives in which the development of shared ownership and collective responsibility serves as a precursor to broader organizational and policy change [15,16,17].

Importantly, the outcomes reported in this study represent process outcomes, implementation outcomes, and systems-learning outcomes rather than clinical or population-level health outcomes. Although the initiative generated evidence supporting the feasibility and acceptability of mobile healthcare delivery and demonstrated measurable growth in coalition development and collaborative infrastructure, it was not designed to evaluate long-term health outcomes, healthcare utilization, housing stability, or mortality. Future research should examine whether the systems infrastructure developed through this initiative ultimately contributes to improvements in healthcare access, continuity of care, service utilization, and health outcomes among people experiencing homelessness.

Despite substantial progress, several challenges remain. Participating organizations identified ongoing needs related to sustainable funding, workforce capacity, governance development, referral coordination, documentation processes, and policy alignment. Coalition members also recognized that sustaining homeless health initiatives will likely require organizational policies, strategic priorities, and operational structures that reinforce commitment beyond individual champions or grant-funded projects. Emerging discussions regarding coalition formalization, shared governance models, and the development of a regional Community of Practice reflect recognition that long-term sustainability depends upon continued investment in collaborative infrastructure and organizational learning [17].

Taken together, the Burke County experience suggests that meaningful improvements in healthcare access for people experiencing homelessness may require communities to invest simultaneously in relationships, governance, service delivery, and systems infrastructure. While the specific strategies described in this report were shaped by the context of a rural Appalachian community, the underlying principles of coalition development, community engagement, shared ownership, human-centered design, and iterative systems learning may offer valuable lessons for other rural communities seeking to strengthen healthcare access for individuals experiencing homelessness.

## 6. Limitations

Several limitations should be considered when interpreting the findings of this project report. This analysis reflects a single rural county context and may not generalize well to other settings, particularly given variation in governance structures, healthcare infrastructure, and available community resources across rural regions. However, the processes described in this report, including coalition building, co-design, and iterative implementation, are not context-dependent interventions but system-level strategies that may be adaptable across diverse settings. As such, while the specific operational model may not be directly replicable, the underlying approach to systems development offers transferable insight for other jurisdictions seeking to improve healthcare access for individuals experiencing homelessness.

In addition, this project emphasizes systems development and implementation learning rather than individual-level clinical outcomes. The analysis does not assess health outcomes, service utilization, or long-term patient impact. The Women’s Health Mobile Unit pilot was intentionally designed as a feasibility and implementation effort to test partnership structures, workflows, and service delivery in a real-world setting. While this approach was appropriate for early-stage system development, assessment of clinical effectiveness and population-level impact will ultimately be needed. Future work should build on this foundation by incorporating outcome evaluation to better understand the effects of mobile and street-based care on health and healthcare utilization.

Finally, the data used in this report were derived primarily from internal project documentation, meeting records, and implementation materials generated by participating organizations. Although these sources provide detailed insight into the evolution of the initiative, they may reflect the perspectives and priorities of project partners. The absence of systematically collected patient-level data or formal qualitative interviews with community members limits the ability to fully capture community perception and lived experience. Future evaluations that incorporate patient-level outcomes, structured stakeholder interviews, and community-based participatory approaches would strengthen understanding of both implementation impact and community relevance.

## 7. Implications for Practice and Policy

The Burke County Homeless Health Initiative provides several practical insights for rural communities seeking to improve healthcare access for individuals experiencing homelessness. First, this project demonstrates that sustainable progress depends less on the development of individual programs and more on collaborative infrastructure that enables coordinated system functioning. Investments in coalition building, shared governance, and cross-sector communication are therefore foundational, not supplemental, to effective service delivery in rural homeless health initiatives.

Second, the experience in Burke County reinforces the value of incorporating HCD approaches into program development. Human-centered design methods ensured that services were informed by the priorities and lived experiences of individuals most affected by gaps in care. This approach extends beyond consultation by positioning community members as active contributors to system design, improving the relevance, acceptability, and responsiveness of services while strengthening trust between providers and underserved populations.

The initiative also demonstrates the value of integrating mobile healthcare strategies within existing community systems rather than implementing them as stand-alone outreach efforts. Alignment with public health operations, healthcare providers, social service organizations, and emergency preparedness structures allowed the initiative to function as part of the local safety-net infrastructure. This level of integration supports continuity of care, improves coordination, and enhances sustainability. Policymakers and funders seeking to support rural homeless health efforts should prioritize models that strengthen cross-sector alignment rather than isolated service delivery.

Despite these advances, long-term sustainability will depend on addressing persistent structural barriers related to financing and workforce capacity. Expanding reimbursement mechanisms for mobile and community-based healthcare delivery, supporting the integration of community health workers and peer support roles, and aligning Medicaid policy with outreach-based models of care are critical next steps. Without these changes, mobile health initiatives will remain dependent on time-limited funding and fragmented implementation structures. Strengthening policy alignment with community-based care delivery models is essential to ensuring that systems developed through local innovation can be sustained and scaled over time.

## 8. Conclusions

This project report describes the evolution of a rural, cross-sector homeless health initiative from an outbreak-driven response to a structured, community co-designed model for healthcare delivery. Findings highlight that while street-based care can improve access and trust among unsheltered populations, its sustainability depends on intentional systems development, including shared governance, aligned partnerships, and integration across the safety-net ecosystem. The Burke County experience demonstrates that public health agencies can play a critical role in convening partners, facilitating coalition development, and guiding system-level transformation beyond direct service provision. Iterative co-design and pilot implementation provided a practical mechanism for translating community-identified needs into actionable service models while strengthening coordination across sectors. These findings suggest that sustainable rural homeless health strategies require movement away from isolated, short-term interventions toward coordinated systems that embed outreach, clinical care, and social services within a shared infrastructure. Future efforts should focus on scaling integrated models, strengthening data systems, and aligning funding mechanisms to support long-term implementation and evaluation in rural settings.

## Figures and Tables

**Table 1:** Pilot Feasibility, Questions, and Outcomes.

Does the Service Meet an Unmet Need?		
Indicator	Questions	Source and Findings
Need (Appropriateness)	What health issues were addressed? Which were not addressed?	Source: Interviews with clients and providers. Findings: Services met most of the needs of women who sought services. Lab services were not initially available.
Satisfaction-client (acceptability)	How did clients feel about the service—responsive to needs, timing, location, scope, format, provider?	Source: Interviews with clients. Findings: Demand was modest, due in part to limited awareness raising and the timing before BUCM was fully operational. The clients that were seen expressed appreciation for the availability of care, the services they received, and the ‘extra’ resources (e.g., care kits, winter clothing).
Satisfaction-Provider (Acceptability)	How did providers feel about the service—timing, location, scope, format, clients, their readiness?	Source: Provider interviews. Findings: Providers (3 in the pilot) consistently voiced that the service model (mobile women’s health) was acceptable
Satisfaction-Community member (Acceptability)		Source: Interviews with clients, observations. Findings: Acceptability was high among users but the mismatch with service timing and transportation suggests this can be improved.
Does the service complement/add value to the current service array?		
Referrals		Source: Service records. Findings: Referrals were made for women served and three men who approached the care team.
Continuity of care		Source: Not measured. Findings: This was not the focus of the pilot but something to explore once/if a sustained service is initiated.
Provider readiness (feasibility)	Did the provider feel prepared and capable of providing BSM?	Source: Provider interviews pre-, during, and post pilot. Findings: Providers consistently described the services as feasible within the setting. Key concerns were primarily focused on process and material availability, all of which were addressed before and during service.
Service Readiness (feasibility)	Did the service have the material resources necessary to provide BSM? Per the plan? Per the requested services/need?	Source: Provider interviews and service records. Findings: Basic care resources were available. Most services were more primary care than women’s health specific.
Is the service feasible in Burke County?		
Site Considerations	Did community members know about the service?	Source: Observation. Fining: Services were marketed pre- and during the pilot through flyers distribution across Morganton and through communications to key service providers. Clients were also encouraged to spread the word and bring peers. Overall, awareness was moderate to low.
	Was the site accessible for clients? Was the site safe for clients?	Source: Observation. Findings: Site was accessible though limited afternoon transportation was an issue. Site safety was generally good though one afternoon a man was expressing threats/concerning behavior in the vicinity. Development and clear and feasible security protocols is recommended.
	Was the site adequate for mobile health care? Was the site safe for providers?	Source: Observation. Findings: Site was accessible to providers, and the mobile care unit was accessible for the planned services. Site safety was generally good (see above).
	Was the site acceptable to the community?	Source: Observation. Findings: The site was acceptable but the afternoon timing was a problem. Now that BUCM is fully operational, this may be less of an issue. Improved planning with future hosts can improve the match between service timing and client availability
Reach	How many different communities were served?	Source: Service records. Findings: Only the BUCM site hosted the service.
Fidelity	Was the service provided within established standards of clinical care? Within the program principles (respect, autonomy)?	Source: Service records, discussions, and observation. Findings: UNC Blue ridge, Health Department and Good Samaritan providers and staff maintained the same high standard of care—quality and respectful—found at their respective agencies.
Systems linkages	Did partners feel informed/in the loop about the service. Did partners feel included in-service operations?	Source: Partner Interviews. Findings: Improvement needed. Continuous communication and inclusiveness during planning for future efforts can contribute to greater ownership and participation. For example, BUCM was not as integrated in service design as they could have been, especially given their centrality in the care system for people experiencing homelessness.
Data system	What worked for collecting and sharing data?	Source: Service Records and observation. Findings: Partners were creative and flexible in learning to use the HD EHR. Data was collected in various ways—impromptu interviews, conversations, team calls, and document review. A more systematic, shared approach will better serve future efforts.
	What did not work?	Source: Service Records and Observation. Timing was a major challenge, with BUCM typically closed before the mobile service arrived. The lack of lab services (due to delayed funding arrangements) was a barrier to the offering of the intended array of services. Community outreach and marketing did not appear to be adequate in scope or early enough to build demand.

## Data Availability

Program documentation and workshop materials are maintained by Burke County Public Health and may be made available upon request to the corresponding author and with permission from Burke County Public Health, subject to confidentiality protections.
